# Development and Validation of 2D-LiDAR-Based Gait Analysis Instrument and Algorithm

**DOI:** 10.3390/s21020414

**Published:** 2021-01-08

**Authors:** Seongjun Yoon, Hee-Won Jung, Heeyoune Jung, Keewon Kim, Suk-Koo Hong, Hyunchul Roh, Byung-Mo Oh

**Affiliations:** 1 Dyphi Research Institute, Dyphi Inc., Daejeon 34068, Korea; seongjun@dyphi.com; 2 Department of Internal Medicine, Seoul National University Hospital, Seoul 03080, Korea; hwjung@amc.seoul.kr; 3 Division of Geriatrics, Department of Internal Medicine, Asan Medical Center, University of Ulsan College of Medicine, Seoul 03080, Korea; 4 Department of Rehabilitation Medicine, National Traffic Injury Rehabilitation Hospital, Gyeonggi-do 12564, Korea; heeyoune@ntrnu.or.kr (H.J.); glacialspike@naver.com (S.-K.H.); 5 Department of Rehabilitation Medicine, Seoul National University Hospital, Seoul National University College of Medicine, Seoul 03080, Korea; keien1@snu.ac.kr

**Keywords:** frailty, gait, physical performance, sarcopenia, LiDAR

## Abstract

Acquiring gait parameters from usual walking is important to predict clinical outcomes including life expectancy, risk of fall, and neurocognitive performance in older people. We developed a novel gait analysis tool that is small, less-intrusive and is based on two-dimensional light detection and ranging (2D-LiDAR) technology. Using an object-tracking algorithm, we conducted a validation study of the spatiotemporal tracking of ankle locations of young, healthy participants (*n* = 4) by comparing our tool and a stereo camera with the motion capture system as a gold standard modality. We also assessed parameters including step length, step width, cadence, and gait speed. The 2D-LiDAR system showed a much better accuracy than that of a stereo camera system, where mean absolute errors were 46.2 ± 17.8 mm and 116.3 ± 69.6 mm, respectively. Gait parameters from the 2D-LiDAR system were in good agreement with those from the motion capture system (r = 0.955 for step length, r = 0.911 for cadence). Simultaneous tracking of multiple targets by the 2D-LiDAR system was also demonstrated. The novel system might be useful in space and resource constrained clinical practice for older adults.

## 1. Introduction

The clinical importance of assessing physical performance is increasing with the global ageing population [1,2]. In older people, the spectrum of physical performance has been studied for its importance in numerous aspects, such as biomarkers of clinical outcome prediction [3], criteria for selecting populations eligible for certain intervention programs [4], and outcome measures per se [5]. Given the clinical relevance, items of physical performance are considered key components in assessing common geriatric syndromes, including frailty and sarcopenia [1].

In addition, various factors of physical performance such as muscle strength, muscle power, balance, and gait parameters have also been extensively studied [6,7]. For example, usual gait speed has been studied for its association with life expectancy [8], risk of fall [9], and risk of adverse outcomes after various clinical procedures [10]. Furthermore, other gait parameters such as cadence, step length, and step width have been also shown to be associated with clinical features, including neurocognitive performance [11], extents of vascular aging, and other geriatric parameters [12,13].

Albeit with clinical importance, acquiring gait parameters other than usual gait speed that can be measured by a stopwatch has been a challenging task in both clinical practice and research focusing on older adults. To address this issue, studies have used both wearable and non-wearable sensors to assess gait parameters either in clinical laboratories or in residential settings [14,15,16]. Using non-wearable sensors, to measure step length, step width, and cadence, subjects were required to walk through walkways with pressure sensor arrays [17] or to undergo marker-based motion capture studies [18], both of which are not highly accessible in most geriatric practices even in developed countries. Furthermore, inviting people to facilities with these instruments might be not a feasible option since many geriatric populations reside in long-term care facilities or communities with decreased mobility. On the other hand, gait analysis using wearable sensors has been extensively studied in research settings [19,20], even though there are still only a few clinically available, standardized sensors used in practice. Therefore, simple, portable sensor-based gait analysis protocols may improve difficulties in studying gait parameters in the older frail population.

For measuring usual gait speed, we previously developed sensor-based instruments and showed cross-correlations between modalities including 1D light detection and ranging (LiDAR), infrared, ultrasound, and laser sensors [21]. Recognizing the advantages of LiDAR technology with its non-intrusiveness and small footprint, in previous studies, we developed and validated a novel algorithm and instrument using a commercially available two-dimensional light detection and ranging (2D-LiDAR) sensor to acquire gait parameters of step length, step width and cadence as well as gait speed. In this study, we aimed to validate this novel software algorithm and instrument with the 3D, marker-based motion capture device as a gold standard method.

## 2. Materials and Methods

### 2.1. Sensors and Installation in Test Environment

**Figure 1:**
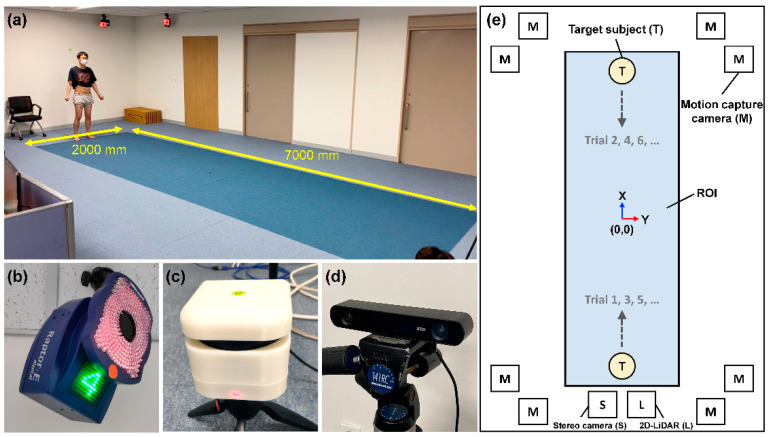
Test environment and instrument settings for validation of two-dimensional light detection and ranging (2D-LiDAR)-based gait analysis. (**a**) Gait analysis room showing region of interest (ROI) area of motion capture system marked as blue on the floor. (**b**) One of eight near infrared cameras of motion capture system. (**c**) 2D-LiDAR sensor. (**d**) Stereo camera. (**e**) A schematic of a test environment with spatial coordinates based on motion capture system. The center of ROI is denoted as (0 mm, 0 mm). The sensor positions of 2D-LiDAR and stereo camera are shown simultaneously.

As a validation target, a commercial 2D-LiDAR sensor (RPLiDAR A3M1, Shanghai Slamtec Co., Ltd., Shanghai, China) with a scan rate of 10–15 Hz and an angular resolution of 0.225° was used (Figure 1c, the sensor is enclosed in a self-designed outer package). Portability (41 mm in height, 76 mm in width, and 190 g in weight), cost-effectiveness (USD ~600), and most importantly, non-intrusiveness of the sensor are attractive characteristics for gait analysis in clinics. The maximum distance ranges from sensor specification were 25 m and 10 m for white and dark objects, respectively. In our study, however, typical sensing ranges were limited to 10 m or closer for a reliable detection, accounting for decreased spatial resolution at longer distances.

A motion capture system (Raptor-E, Motion Analysis, USA) was regarded as a gold standard method to acquire reference gait data in this study. The system consists of eight near-infrared (750 nm) cameras with a frame size of 1.3 megapixels and a resolution of 1280 × 1024 pixels (Figure 1a,b). The frame rate was set to 60 frames per second (fps). To determine joint positions, additional attachment of infra-red reflective markers on the human body is necessary. A total of 15 markers were attached at the lower body of each human subject (see detailed marker placement in Figure S1. In particular, spatiotemporal locations of left and right ankle markers were used for gait analysis in this study.

As a potential alternative to the 2D-LiDAR sensor, a commercial stereo camera (ZED2, Stereolabs Inc., USA) was also included in this study (Figure 1d). The stereo camera, which is portable, cost-effective (USD ~449), and non-intrusive like 2D-LiDAR, estimates depths of objects in a scene based on a stereo-matching method. Therefore, the stereo camera sensor accompanied the gait motion tracking to evaluate the performances of both sensors. The field of view (FoV) of the stereo camera sensor was 110° (horizontal) × 70° (vertical) × 120° (depth) and the frame rate was set to 30 fps.

To validate the gait monitoring instrument based on the 2D-LiDAR sensor, all tests were performed at a dedicated gait analysis laboratory at the National Traffic Injury Rehabilitation Hospital, South Korea. A paired motion capture camera was installed at each corner of the laboratory room (see Figure 1e, a single motion capture camera is denoted as M), which forms a region of interest (ROI) of the entire motion capture system. Though the full ROI for motion capture system was 7000 × 2000 mm^2^ (see Figure 1a, colored by dark blue on the floor), in this study, the area of 6000 × 2000 mm^2^ in the middle of the ROI was mainly monitored to ensure reliable data acquisition from the motion capture system. One 2D-LiDAR and one stereo camera sensor were located at the one end side of ROI (denoted as L and S in Figure 1e). The heights of the 2D-LiDAR and stereo camera sensors were empirically determined for the best acquisition of spatiotemporal data of left and right ankles during gait motions. Since the 2D-LiDAR sensor can only detect a two-dimensional spatial plane, the height of the 2D-LiDAR sensor was adjusted to detect the ankles of human subjects. On the other hand, the height of the stereo camera sensor was adjusted to around the height of the trunks of the human subjects, since the stereo camera sensor acquires three-dimensional spatial data.

### 2.2. Participants

For the tests, four healthy participants were enrolled (male, aged 30–45). The participants were guided to walk through the forward and reverse directions along the *x*-axis in each trial (see the walking directions in Figure 1e and Supplementary Video 1). A total of 40 trials, 10 trials per participant, were conducted. The institutional review board of the National Traffic Injury Rehabilitation Hospital approved the study protocol (No. NTRH-20004), and written informed consent was acquired from all participants.

### 2.3. Object Tracking Algorithm for 2D-LiDAR Sensor

**Figure 2:**
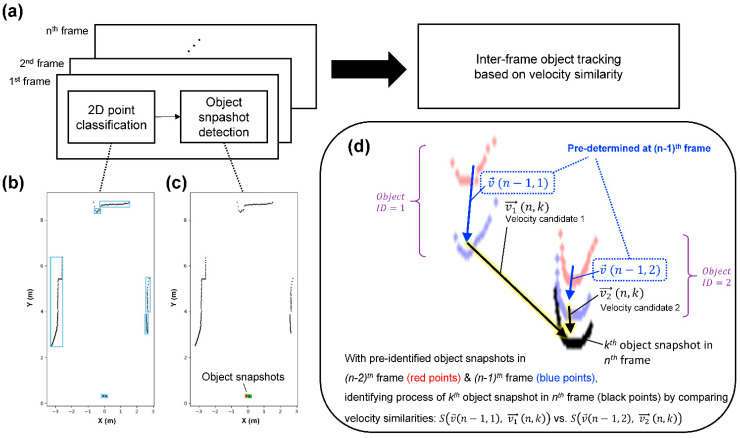
Concept of object tracking algorithm for 2D-LiDAR sensor. (**a**) Simplified flow chart of inertia-based object tracking algorithm (IOTA) (see Supplementary Figure S13 for full version). (**b**) Point groups represented as bounding boxes after point classification. (**c**) Point groups are filtered out by object size and curvature criteria to remove background. (**d**) An example case of inter-frame object tracking where probable velocity candidates, $\vec{v_{1}}\left(n,k\right)$ and $\vec{v_{2}}\left(n,k\right)$, of the *k*th object snapshot in the *n*th frame are compared to the predetermined velocities, $\vec{v}\left(n-1,\;1\right)$ and $\vec{v}\left(n-1,\;2\right)$, of object ID 1 and 2 in the (*n* − 1)th frame.

In this study, a novel object tracking algorithm, namely, the inertia-based object tracking algorithm (IOTA), was developed to recognize left and right ankles from raw point cloud data of the 2D-LiDAR sensor and to track spatiotemporal locations of the ankles (Figure 2a). IOTA is based on the assumption that the velocity of slowly moving objects is negligibly changed if data scan rates are fast. Therefore, IOTA tracks objects by comparing similarities in inter-frame velocities. For each 2D-LiDAR scanning frame, the points in raw point cloud data are classified as point groups by nearest neighbor clustering, and subsequently, object snapshots are detected by filtering the point groups with size and curvature criteria, as shown in Figure 2b,c respectively (see the flow chart in Figure S13). Once object snapshots in current frame are determined, by comparing to object velocities in the previous frame, Euclidean distance scores (*S*) of velocities for each object snapshot in the current frame, representing the velocity similarities, are calculated as follows:

(1) $$S\left(n,k,m\right)=\frac{1}{1+d\left(\vec{v_{m}}\left(n,k\right),\;\vec{v}\left(n-1,\;m\right)\right)}$$

where $\vec{v_{m}}\left(n,k\right)$ is the velocity of *k*th object snapshot in the *n*th frame, assuming that the object snapshot corresponds to object ID = *m* in the previous frame, $\vec{v}\left(n-1,\;m\right)$ is the velocity of the object ID = *m* in the previous frame, and $d\left(\vec{v_{m}}\left(n,k\right),\;\vec{v}\left(n-1,\;m\right)\right)$ is the Euclidean distance between the two velocities. Consequently, the object ID of the *k*th object snapshot in the current *n*th frame is determined by selecting *m* giving the maximum *S*.

### 2.4. Object Tracking Algorithm for Stereo Camera

For the stereo camera, the spatiotemporal locations of the left and right ankles were tracked by combining the 2D human pose estimation (2D-HPE) network with depth data gathered from the stereo camera. The 2D-HPE network was applied to the left camera image for each frame. In this study, a well-trained 2D-HPE network provided by openVINO toolkit (Intel Corp.) was selected [22]. Although the estimated pose contained up to 18 key points, only the key points of left and right ankles were used. The representative pixels of the left and right ankles were converted to 3D points by depth maps corresponding to the pixel maps.

### 2.5. Performance Evaluation Methods for Object Tracking and Gait Parameters

Performances of 2D-LiDAR with IOTA and stereo camera with 2D-HPE were evaluated by comparing spatiotemporal tracking results to those of the motion capture system for each trial. The mean absolute error (MAE) was used to represent the tracking accuracies of each modality. The step length, step width, cadence, gait speed, stride length, stride time, step time, swing time, and stance time are derived as secondary gait parameters from the spatiotemporal tracking results of ankles. The accuracies of the gait parameters from 2D-LiDAR with IOTA and stereo camera with 2D-HPE were evaluated by regression and Bland–Altman analysis.

## 3. Results

### 3.1. Spatiotemporal Tracking of Ankle Locations

**Figure 3:**
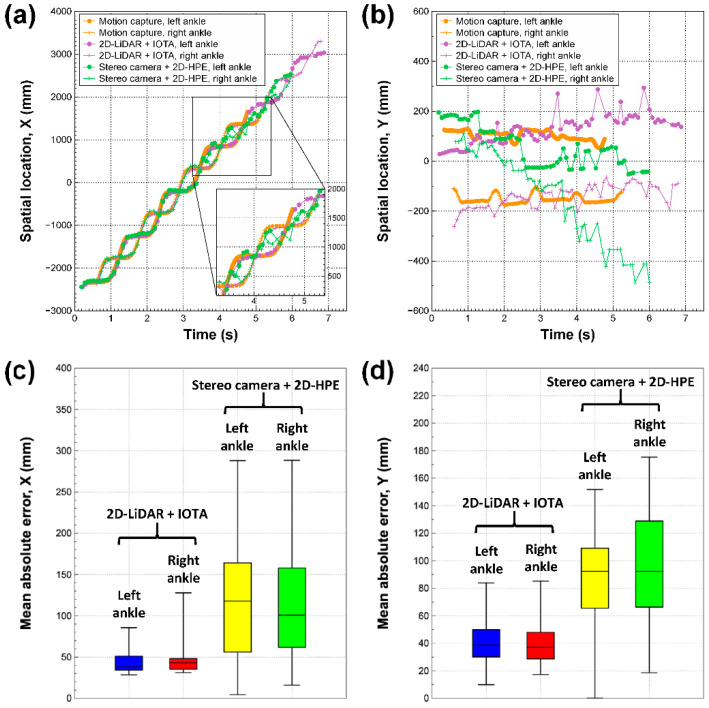
Ankle tracking results and accuracy via 2D-LiDAR with IOTA and stereo camera with 2D human pose estimation (2D-HPE). (**a**,**b**) Representative tracking results of spatiotemporal locations of ankles along the walking direction (**a**) and the lateral direction (**b**), gathered by motion capture system, 2D-LiDAR with IOTA, and stereo camera with 2D-HPE during the first trial of subject 4 (the entire tracking results are shown in Figures S5–S12). The sensor positions of 2D-LiDAR and stereo camera are at x = −4000 mm. (**c**,**d**) Mean absolute errors of the spatial locations for 2D-LiDAR with IOTA and stereo camera with 2D-HPE along the walking direction (**c**) and along the lateral direction (**d**), respectively.

For three different modalities of motion capture system, 2D-LiDAR with IOTA, and the stereo camera with 2D-HPE, the tracked spatiotemporal locations of ankles along the axial (*x*-axis) and lateral (*y*-axis) directions of walking are shown in Figure 3a,b respectively. Regarding the tracking results of the motion capture system as ground truths, Figure 3c,d show the mean absolute error (MAE) of the spatial locations of ankles along the axial and lateral directions to the walking direction, respectively, for 2D-LiDAR with IOTA and the stereo camera with 2D-HPE. The average MAEs for left/right ankles on 2D-LiDAR with IOTA and stereo camera with 2D-HPE were 45.1 ± 16.3 mm/47.3 ± 19.4 mm and 114.5 ± 66.3 mm/118.1 ± 73.6 mm, respectively, along the axial direction (Figure 3c). The low mean values and shallow distributions in MAE on 2D-LiDAR with IOTA are clearly seen. In the lateral direction, the average MAEs were 40.6 ± 16.7 mm/40.6 ± 16.5 mm and 87.8 ± 36.8 mm/96.7 ± 38.8 mm on 2D-LiDAR with IOTA and the stereo camera with 2D-HPE, respectively (Figure 3d). The tracking accuracy of 2D-LiDAR with IOTA was mostly affected by the low frame rate (10–15 fps) of the sensor and occlusion events. Since IOTA is based on the assumption that the inter-frame velocities would be similar, increasing the inter-frame intervals caused by the low frame rate or the occlusion events, where object snapshots are blinded in a certain interval, eventually diminishes the velocity similarity. On the other hand, since the object tracking of the stereo camera with 2D-HPE is solely performed in a single frame, the tracking error was mostly caused by inaccuracies in pose estimation and depth sensing. Even a slight error in the 2D keypoint locations from the pose estimation induced a significant tracking error when the 2D pixel position was unprojected to 3D spatial domain combined with a depth map. The depth fluctuation, which is a fundamental limitation of stereo camera due to feature-based depth estimations, was further deteriorated the tracking accuracy.

In addition, compared to the tracked spatial locations of ankles along the axial direction (Figure 3a), the spatial locations along the lateral direction showed poor agreement with those of the motion capture system for both of 2D-LiDAR with IOTA and the stereo camera with 2D-HPE (Figure 3b), because of the limited spatial resolutions of each sensor. Slight mismatches between the walking axis (i.e., y = 0), and the sensor positions (see Figure 1e) also affected the spatiotemporal accuracy, especially of the stereo camera with 2D-HPE, where a significant spatial drift was observed, while 2D-LiDAR with IOTA is much more tolerant to the spatial drift, as shown in Figure 3b.

### 3.2. Correlations Between Gait Parameters Derived from Three Different Modalities

**Figure 4:**
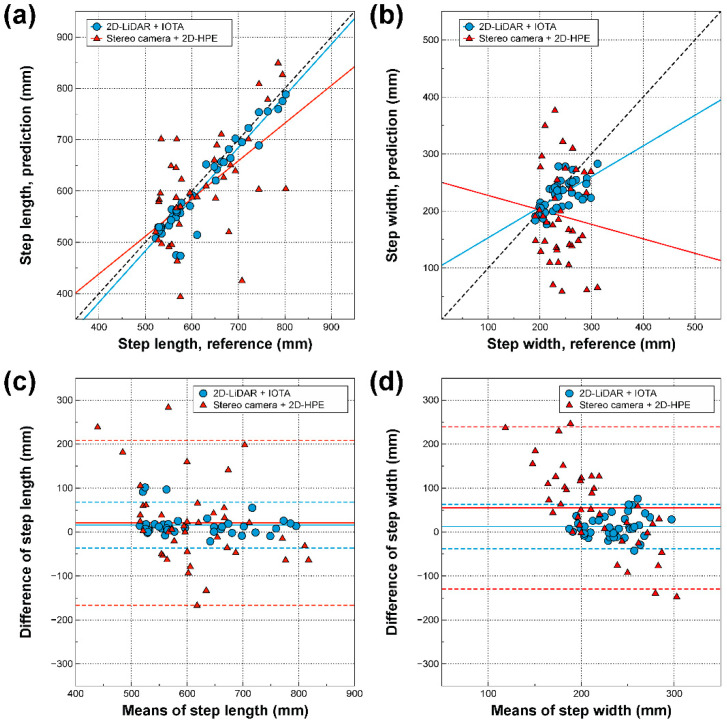
Step length and width derived from spatiotemporal locations of tracked ankles via 2D-LiDAR with IOTA and stereo camera with 2D-HPE. (**a**,**b**) Regression and (**c**,**d**) Bland-Altman plots with motion capture system for 2D-LiDAR with IOTA and stereo camera with 2D-HPE for step length (**a**,**c**) and step width (**b**,**d**). The black dotted lines in the regression plots are ideal lines for perfect correlations. In the Bland–Altman plots, each solid horizontal line represents the bias of each modality. The upper and lower dashed horizontal lines from each bias represent 95% confidence intervals.

**Figure 5:**
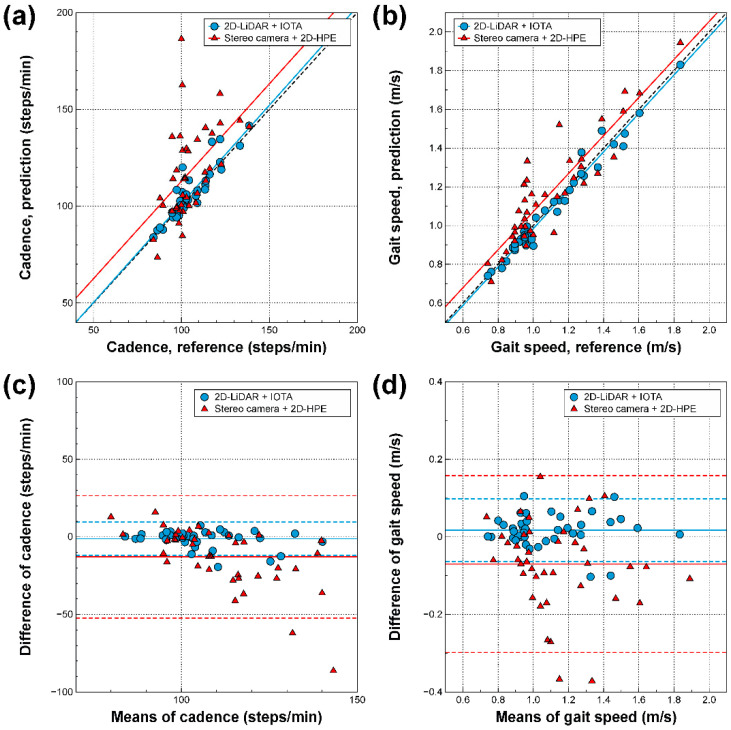
Cadence and gait speed derived from spatiotemporal locations of tracked ankles via 2D-LiDAR with IOTA and stereo camera with 2D-HPE. (**a**,**b**) Regression and (**c**,**d**) Bland–Altman plots with motion capture system for 2D-LiDAR with IOTA and stereo camera with 2D-HPE for cadence (**a**,**c**) and gait speed (**b**,**d**). The black dotted lines in the regression plots are ideal lines for perfect correlations. In the Bland–Altman plots, each solid horizontal line represents the bias of each modality. The upper and lower dashed horizontal lines from each bias represent 95% confidence intervals.

The regressions and Bland–Altman plots for various gait parameters derived from the spatiotemporal locations of ankles on 2D-LiDAR with IOTA and the stereo camera with 2D-HPE, where the gait parameters from the motion capture system were regarded as references, are shown in Figure 4 and Figure 5. Step length, step width, cadence, gait speed, and other spatial and temporal gait parameters were also extracted, as shown in the Supplementary Materials (Figures S2–S4). As shown in Figure 4a and Figure 5a, strong correlations are seen between 2D-LiDAR with IOTA and the motion capture system for step length (*r* = 0.955, *p* < 0.001) and cadence (*r* = 0.911, *p* < 0.001), while the correlations between the stereo camera with 2D-HPE and motion capture system are weaker for step length (r = 0.555, *p* < 0.001) and cadence (*r* = 0.510, *p* < 0.001). The Bland–Altman plot in Figure 4c and Figure 5c also showed a narrower error distribution for 2D-LiDAR with IOTA than for the stereo camera with 2D-HPE. For step width, on the other hand, weak correlations are observed for both 2D-LiDAR with IOTA (*r* = 0.623, *p* < 0.001) and the stereo camera with 2D-HPE (*r* = −0.098, *p* = 0.551), as shown in Figure 4b, since the spatial accuracy along the lateral direction was poor (Figure 3b). For gait speed, the strongest correlations were observed for both of 2D-LiDAR with IOTA (*r* = 0.986, *p* < 0.001) and the stereo camera with 2D-HPE (*r* = 0.903, *p* < 0.001), as shown in Figure 5b, where the errors are mostly within 95% confidence intervals (Figure 4d). It is also noteworthy that the bias in 2D-LiDAR with IOTA was close to zero (Figure 4c,d and Figure 5c,d), while the bias in stereo camera with 2D-HPE shifted from zero for some cases (Figure 4d and Figure 5c,d).

### 3.3. Demonstration of Multiple Target Tracking by 2D-LiDAR with IOTA

**Figure 6:**
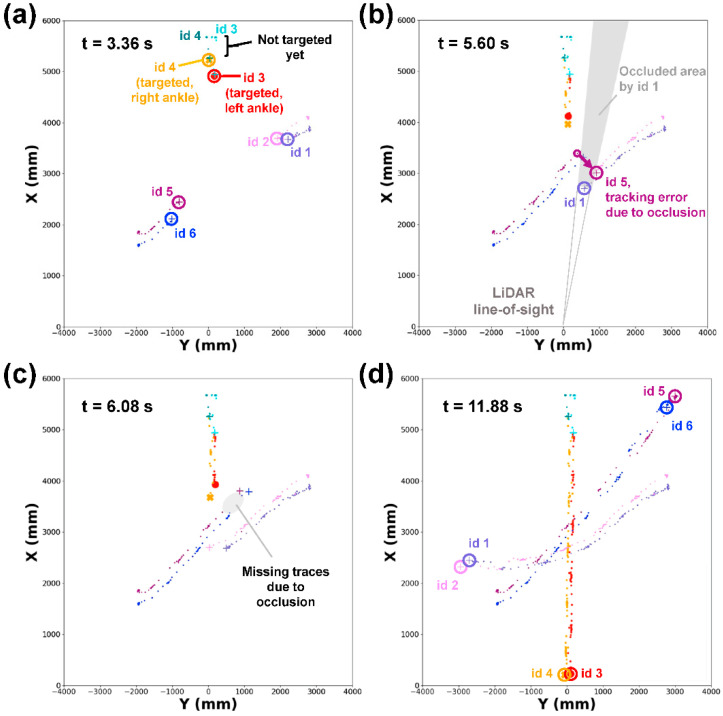
Tracking test for multiple targets via 2D-LiDAR with IOTA. (**a**–**d**) Tracked object snapshots for various time frames with the initial detection of the target ankles (**a**), the occurrence of occlusion (**b**), the restoration of tracking from the occlusion (**c**), and the end of measurement (**d**). A total of three people (i.e., six feet) with the targeted left (ID 3) and right (ID 4) ankles, and untargeted ankles (ID 1, 2, 5, and 6) are presented.

Since 2D-LiDAR sensor collects spatiotemporal information inherently in a non-intrusive manner, we assessed the expandability of 2D-LiDAR with IOTA to track multiple targets without any instrumental modifications. Figure 6 shows an example of multi-target tracking to mimic a gait monitoring situation where one subject is targeted while two unwanted people are crossing the monitoring region (see the test environment and walking of subjects in Video S2). Before the subject started to walk (t < 2.20 s), the ankles of the subject were still being tracked but not targeted because the left and right ankles were not yet determined (non-targeted object ID three and four in Figure 6a). During the initial movement of object snapshots with ID three and four (i.e., the subject started to walk), the left and right ankles were determined automatically (t < 3.36 s). Meanwhile, the other object snapshots were being tracked concurrently (non-targeted ID one, two, five, and six in Figure 6a). After determining the target ankles, the target object IDs were remembered. During the entire gait monitoring test, a few occlusion events occurred. Figure 6b,c show an example case of the occlusion events, where the object snapshots with ID one and two had concealed the object snapshots with ID five and six from the line-of-sight of 2D-LiDAR sensor, which led to missing the spatiotemporal locations of the object snapshots with ID five and six for some time (~0.5 s). Because of the occlusion, the tracking became erroneous (object ID five in Figure 6b) or halted (missing traces of the objects ID five and six in Figure 6c). Nonetheless, once the object snapshots appeared again, the tracking was successfully restored with appropriate object IDs because IOTA assesses the most probable matching of the object snapshots based on the inertia (Figure 6c). Similarly, for the targeted ankles (i.e., targeted object ID three and four), two occlusion events were inevitable where the occlusions due to the object snapshots with ID five and six were the first, followed by object snapshots with ID one and two. Despite the occlusions, tracking of the targeted ankles was well maintained as presented in walking traces in Figure 6d. A full video clip for the multi-target tracking in Figure 6 is shown in Video S3. Other multi-target tracking tests that simulated various situations, including assisted walk, horizontal walk, and random walk, are also shown in Videos S4–S6.

## 4. Discussion

In this study, we established a small-sized, portable 2D-LiDAR-based gait analysis system that can be used in spaces typically up to ~100 m^2^ to assess gait parameters of multiple people in a non-intrusive manner and found that the accuracy of parameters from the newly developed system was comparable to that of a dedicated motion capture system. To the authors’ knowledge, this study is the first to use 2D-LiDAR to examine human physical parameters of walking.

Specifically, in our study using 2D-LiDAR with IOTA, the spatial locations along the walking direction corresponded with those of the motion capture system, even though the tracked point data were sparse. Since the 2D-LiDAR sensor directly measures absolute distances of objects from the sensor based on a time-of-flight of laser, the measured depth information is highly accurate and reliable. Nevertheless, some inaccuracies in ankle tracking were inevitable at far distances. The main reason for the inaccuracy is a lack of point data at far distances. Because of the limited angular resolution of the 2D-LiDAR sensor, the spatial resolution decreases as the distance from the sensor increases, where we observed that the points almost disappear if the distance between the ankles and the sensor is larger than 10 m.

Similarly, for the stereo camera with 2D-HPE, the tracking accuracy decreases as targets move further away from the sensor. However, the severity and mechanism of decline in accuracy are different in the case of 2D-LiDAR with IOTA. Since the stereo camera estimates the distances of objects from the sensor using the disparity in binocular images, accuracy degradation is inevitable for far objects because of the smaller features and textures of the objects in images. In addition to the intrinsic limitations of a stereo camera, an estimation error from the 2D-HPE network is also crucial for tracking accuracy. As subjects move further away, the number of pixels consisting of the images gradually decreases, i.e., image resolution is reduced, diminishing the essential features for recognizing poses in the 2D-HPE network [23]. Consequently, corresponding pixel outputs indicating each keypoint, including left and right ankles, become erroneous.

With supporting clinical evidence, researchers have been keen to measure gait speed and parameters effectively in clinical practice and in research on older adults. Historically, approaches for acquiring gait parameters have been largely divided under two differing concepts, namely, the use of wearable sensors [18,24] and the use of external devices such as stereo cameras, pressure sensors [17], beam breakers [25], and sophisticated motion capture systems [18]. These systems have specific advantages and drawbacks in terms of widespread use in studying the physical performance of older adults. For example, when used appropriately and for a long-term period, wearable devices using inertial measurement units can provide data on physical activity and usual gait speed in life space [24]. However, research has shown that long-term compliance with wearable devices is poor in the older population [26]. On the other hand, marker-based systems or walkways can only be used in clinical/laboratory settings, permitting the assessment of gait parameters in a cross-sectional manner. While a single, small-sized stereo camera can capture motion parameters, its short range of reliable measurement precludes the acquisition of meaningful lengths of walking, as observed in this study.

With characteristic advantages, 2D-LiDAR with IOTA may fill these gaps in existing modalities. As Piau et al. suggested in a recent study, measuring walking speed using home-embedded infrared sensors could help differentiate between eventual fallers and non-fallers [27]. In a living space, 2D-LiDAR with IOTA may monitor physical activity and physical performance in real time, with wide detection ranges, in a non-intrusive manner and also in a longitudinal manner. Gait instabilities that may be detected in snapshot measurements may be revealed in the real world at times of cognitive burden, such as dual-task situations. In this instance, the relationship between the living space measurement of gait parameters and clinic-based measurement to assess frailty and predict adverse health outcomes might be analogous to the clinic-based 12-lead electrocardiogram and loop recording in approaching syncope [28]. Along with assessing gait parameters per se, monitoring physical performance in the living space may help detect acute exacerbation, deconditioning, and progression of chronic organ diseases such as chronic obstructive pulmonary disease [29,30] or congestive heart failure in older adults [31]. Further prospective, real-world studies using 2D-LiDAR with IOTA can be used to assess the possible clinical benefits of real-time physical performance monitoring in frail multimorbid older adults.

In clinical practice, access to gait analysis is limited in most settings by restraints in space and human resources. The new system may be used to acquire gait parameters even when patients enter the clinic room. This newer, 2D system may resolve some drawbacks of previously reported wall-attached infrared beam breakers that could be used to measure gait speeds of patients in outpatient geriatric clinics [25]. While beam breakers cannot distinguish between the patient and caregivers who often accompany outpatients, 2D-LiDAR with IOTA can assess parameters of multiple persons simultaneously, as we demonstrated in this study. As the present study shows improved accessibility to gait parameters, in the future, the relevance of gait parameters to clinical outcomes in various medical or surgical situations should be assessed.

However, our study had several limitations. Although 2D-LiDAR with IOTA can produce gait parameters comparable to the motion capture system, whole-body motion analysis cannot be predicted with the technology because 2D-LiDAR sensor only gathers depth data in a two-dimensional plane. With the continuous scaling in sensor footprint, the angular resolution and price of LiDAR sensors have been improving rapidly; future studies on IOTA with 3D-LiDAR would be interesting. In this preliminary study of the protocol and initial validation, only young and healthy participants were included. However, since frail older people tend to walk slower than younger people, the spatiotemporal reliability of newly developed protocol is not likely to differ substantially when used in older adults.

## 5. Conclusions

The 2D-LiDAR with IOTA was proposed as a practical gait analysis solution for small and noisy clinical environments. Its tracking ability of left and right ankles was validated by comparing it with the motion capture system, which is the gold standard, and with stereo camera with 2D-HPE as a potential alternative. A good agreement between the 2D-LiDAR with IOTA and motion capture system was observed, where the spatial inaccuracy in MAE was as low as 46.2 ± 17.8 mm, which is much better than 116.3 ± 69.6 mm of the stereo camera with 2D-HPE. The tracking accuracy of 2D-LiDAR with IOTA was affected by frame rate, occlusion, and absolute distance of objects from the sensor. Based on the advantages of the 2D-LiDAR sensor, such as cost-effectiveness, highly accurate depth measurement, small footprint, and simple installation characteristics, 2D-LiDAR with IOTA can be a promising solution for clinical environments, where a simple and quick gait analysis is necessary.

## Data Availability

The data that support the findings of this study are available from the corresponding author upon reasonable request.

## Supplementary Materials

The following are available online at https://www.mdpi.com/1424-8220/21/2/414/s1, Figure S1: Marker positions attached to subjects for gait analysis via motion capture system, Figure S2: Additional spatial gait parameter, Figure S3: Additional temporal gait parameters of stride and step times, Figure S4: Additional temporal gait parameters of swing and stance times, Figures S5–8: Tracked spatiotemporal locations of ankles along the walking direction gathered by motion capture system, 2D-LiDAR with IOTA, and stereo camera with 2D-HPE for the 1st–4th subjects, Figures S9–12: Tracked spatiotemporal locations of ankles along the lateral direction gathered by motion capture system, 2D-LiDAR with IOTA, and stereo camera with 2D-HPE for the 1st–4th subjects, Figure S13: Flow chart of IOTA, Videos S1–S6.
