# Supplementary material for: Development and Validation of 2D-LiDAR-Based Gait Analysis Instrument and Algorithm
Source: Sensors (Basel). 2021 Jan 8;21(2):414. doi: 10.3390/s21020414 (PMC7826665; doi:10.3390/s21020414)
Supplement: Supplementary file 1 [file sensors-21-00414-s001.zip › sensors-1055555-supply/sensors-1055555-supplementary.docx]

Article

Development and Validation of 2D-LiDAR-Based Gait Analysis Instrument and Algorithm

Seongjun Yoon ^1,†^, Hee-Won Jung ^2,3,†^, Heeyoune Jung ^4^, Keewon Kim ^5^, Suk Koo Hong ^4^, Hyunchul Roh ^1,^* and Byung-Mo Oh ^4,5,^*

^1^ Dyphi Research Institute, Dyphi Inc., 34068 Daejeon, Korea; seongjun@dyphi.com

^2^ Department of Internal Medicine, Seoul National University Hospital, 03080 Seoul, Korea; [hwjung@amc.seoul.kr](mailto:hwjung@amc.seoul.kr)

^3^ Division of Geriatrics, Department of Internal Medicine, Asan Medical Center, University of Ulsan College of Medicine, 03080 Seoul, Korea

^4^ Department of Rehabilitation Medicine, National Traffic Injury Rehabilitation Hospital, 12564 Gyeonggi-do, Korea; heeyoune@ntrnu.or.kr (H.J.); glacialspike@naver.com (S.K.H.)

^5^ Department of Rehabilitation Medicine, Seoul National University Hospital, Seoul National University College of Medicine, 03080 Seoul, Korea; keien1@snu.ac.kr

† These authors contributed equally to the work

***** Correspondence: roh@dyphi.com (H.R.); moya1@snu.ac.kr (B.-M.O.)

| **Citation:** Seongjun, Yoon.; Jung, H.-W.; Jung, H.; Kim, K.; Hong, S.K.; Roh, H.; Oh, B.-M. Development and Validation of 2D-LiDAR-Based Gait Analysis Instrument and Algorithm. *Sensors* **2021**, *21*, x. https://doi.org/10.3390/xxxxx  Received: 13 December 2020  Accepted: 6 January 2021  Published: date  **Publisher’s Note:** MDPI stays neutral with regard to jurisdictional claims in published maps and institutional affiliations.  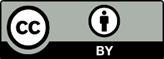  **Copyright:** © 2020 by the authors. Submitted for possible open access publication under the terms and conditions of the Creative Commons Attribution (CC BY) license (http://creativecommons.org/licenses/by/4.0/). |
| --- |

**Figure S1.** Marker positions attached to subjects for gait analysis via motion capture system.Spatiotemporal marker locations of L.Ankle and R.Ankle were used for gait analysis in the study.

**Figure S2.** Additional spatial gait parameter (a) Regression and (**b)** Bland-Altman plots with motion capture system for 2D-LiDAR with IOTA and stereo camera with 2D-HPE for stride length. The black dotted line in the regression plot is the ideal line for perfect correlation. In the Bland-Altman plot, each solid horizontal line represents the bias of each modality. The upper and lower dashed horizontal lines from each bias represent 95% confidence intervals.

**Figure S3.** Additional temporal gait parameters of stride and step times **(a-b)** Regression and (**c-d)** Bland-Altman plots with motion capture system for 2D-LiDAR with IOTA and stereo camera with 2D-HPE for (**a, c)** stride time and (**b, d)** step time. The black dotted lines in the regression plots are ideal lines for perfect correlations. In the Bland-Altman plots, each solid horizontal line represents the bias of each modality. The upper and lower dashed horizontal lines from each bias represent 95% confidence intervals.

**Figure S4.** Additional temporal gait parameters of swing and stance times **(a-b)** Regression and (**c-d)** Bland-Altman plots with motion capture system for 2D-LiDAR with IOTA and stereo camera with 2D-HPE for (**a, c)** swing time and (**b, d)** stance time. The black dotted lines in the regression plots are ideal lines for perfect correlations. In the Bland-Altman plots, each solid horizontal line represents the bias of each modality. The upper and lower dashed horizontal lines from each bias represent 95% confidence intervals.

**Scheme S5.** Tracking test for multiple targets via 2D-LiDAR with IOTA a-d Tracked object snapshots for various time frames with the initial detection of the target ankles **(a)**, the occurrence of occlusion **(b)**, the restoration of tracking from the occlusion **(c)**, and the end of measurement **(d)**. Total three persons (i.e., six feet) with the targeted left (ID 3) and right (ID 4) ankles, and untargeted ankles (ID 1, 2, 5, and 6) are presented.

Demonstration of multiple target tracking by 2D-LiDAR with IOTA

Since 2D-LiDAR sensor collects spatiotemporal information inherently in a non-intrusive manner, we assessed the expandability of 2D-LiDAR with IOTA to track multiple targets without any instrumental modifications. Supplementary Figure S5 shows an example of multi-target tracking to mimic a gait monitoring situation where one subject is targeted while two unwanted persons are crossing the monitoring region (see the test environment and walking of subjects in Supplementary Video 2). Before the subject started to walk (t < 2.20 s), the ankles of the subject were still being tracked but not targeted because the left and right ankles was not yet determined (non-targeted object ID 3 and 4 in Supplementary Figure S5a). During the initial movement of object snapshots with ID 3 and 4 (i.e., the subject started to walk), the left and right ankles were determined automatically (t < 3.36 s). Meanwhile, the other object snapshots were being tracked concurrently (non-targeted ID 1, 2, 5, and 6 in Supplementary Figure S5a). After determining the target ankles, the target object IDs were remembered. During the entire gait monitoring test, a few occlusion events occurred. Supplementary Figures S5b and c show an example case of the occlusion events, where the object snapshots with ID 1 and 2 had concealed the object snapshots with ID 5 and 6 from the line-of-sight of 2D-LiDAR sensor, which led to missing the spatiotemporal locations of the object snapshots with ID 5 and 6 for some time (~0.5 s). Because of the occlusion, the tracking became erroneous (object ID 5 in Supplementary Figure S5b) or halted (missing traces of the objects ID 5 and 6 in Supplementary Figure S5c). Nonetheless, once the object snapshots appeared again, the tracking was successfully restored with appropriate object IDs because IOTA assesses the most probable matching of the object snapshots based on the inertia (Supplementary Figure S5c). Similarly, for the targeted ankles (i.e., targeted object ID 3 and 4), two occlusion events were inevitable where the occlusions due to the object snapshots with ID 5 and 6 were the first, followed by object snapshots with ID 1 and 2. Despite the occlusions, tracking of the targeted ankles was well maintained as presented in walking traces in Supplementary Figure S5d. A full video clip for the multi-target tracking in Supplementary Figure S5 is shown in Supplementary Video 3. Other multi-target tracking tests that simulated various situations, including assisted walk, horizontal walk, and random walk, are also shown in Supplementary Videos 4-6.
